# Utilisation of a mitochondrial intergenic region for species differentiation of fruit flies (Diptera: Tephritidae) in South Africa

**DOI:** 10.1186/s12864-022-09038-x

**Published:** 2022-12-01

**Authors:** Kelsey J Andrews, Rachelle Bester, Aruna Manrakhan, Hans J Maree

**Affiliations:** 1 Department of Genetics, Stellenbosch University, Private Bag X1, 7602 Matieland, South Africa; 2 Citrus Research International, PO Box 2201, 7602 Matieland, South Africa; 3 Citrus Research International, PO Box 28, 1200 Mbombela, South Africa; 4 Department of Conservation Ecology and Entomology, Stellenbosch University, Private Bag X1, 7602 Matieland, South Africa

**Keywords:** Species identification, Mitochondrial DNA, Intergenic spacer, *Ceratitis capitata*, *Ceratitis cosyra*, *Ceratitis rosa*, *Ceratitis quilicii*, *Bactrocera dorsalis*.

## Abstract

**Background:**

Fruit flies (Diptera: Tephritidae) comprise species of agricultural and economic importance. Five such fruit fly species are known to affect commercial fruit production and export in South Africa: *Ceratitis capitata*, *Ceratitis cosyra*, *Ceratitis rosa*, *Ceratitis quilicii*, and *Bactrocera dorsalis*. Management practices for these pests include monitoring, application of pest control products, post-harvest disinfestation measures and inspection of consignments both prior to shipment and at ports of entry. In activities relating to monitoring and inspection, accurate identification of these pests to species level is required. While morphological keys for adult stages of these fruit fly species have been well developed, morphological keys for earlier life stages remain problematic. In instances where closely related species cannot be reliably distinguished morphologically, there is a need for molecular tools to assist in identifying these five fruit fly species during surveillance practices, where sequencing-based approaches would be beneficial.

**Results:**

Two complete mitochondrial genomes were assembled for each fruit fly species investigated using high throughput sequencing data generated in this study. A single primer set was designed to amplify a region between tRNA^ile^ and tRNA^met^. The amplicon consists of a partial segment of tRNA^ile^, intergenic region I (tRNA^ile^ - tRNA^gln^), the complete sequence of tRNA^gln^, intergenic region II (tRNA^gln^ - tRNA^met^), and a partial segment of tRNA^met^. PCR amplicons were generated for 20 specimens of each species, five of which were colony adult males, five colony larvae, and 10 wild, trap-collected specimens. Upon analysis of the amplicon, intergenic region I was identified as the most informative region, allowing for unambiguous identification of the five fruit fly species. The similarity in intergenic region II was too high between *C. rosa* and *C. quilicii* for accurate differentiation of these species.

**Conclusion:**

The identity of all five fruit flies investigated in this study can be determined through sequence analysis of the mitochondrial intergenic regions. Within the target amplicon, intergenic region I (tRNA^ile^ - tRNA^gln^) shows interspecific variation sufficient for species differentiation based on multiple sequence alignment. The variation in the length of intergenic region I is proposed as a potential tool for accurately identifying these five fruit flies in South Africa.

**Supplementary Information:**

The online version contains supplementary material available at 10.1186/s12864-022-09038-x.

## Background

Five fruit fly species in the family Tephritidae (Order Diptera) affect fresh fruit production and export in South Africa [1]. Four of these flies are of Afrotropical origin belonging to the genus *Ceratitis* MacLeay; *Ceratitis capitata* (Wiedemann), the Mediterranean fruit fly; *Ceratitis cosyra* (Walker), the marula fly; *Ceratitis rosa* Karsch, the Natal fly; and *Ceratitis quilicii* De Meyer, Mwatawala & Virgilio, the Cape fly [2]. The latter fruit fly is a newly described species; its current host range and geographic distribution are still being determined [3]. The fifth fruit fly species: *Bactrocera dorsalis* (Hendel), is of Asian origin and invaded the northern areas of South Africa in 2013 [4]. Fruit fly pests cause physical damage to fruit produced in South Africa through oviposition, leaving puncture marks on the skin and decay in the flesh rendering it unmarketable [5, 6]. Other economic damage is incurred from export market restrictions due to the quarantine status and invasion potential of these flies [7–11]. While the five fruit fly species focused on in this study are not the only tephritid pests present in South Africa, they are currently the only fruit fly pests of commercial fresh fruit exported from South Africa. Country-specific phytosanitary certification measures are in place to ensure that consignments containing plant products such as fresh fruit are free from quarantine pests upon arrival at Ports of Entry (PoE) [12, 13]. Export market requirements change constantly based on the absence or presence of pests in both the exporting and importing countries. The European Union (EU) is a significant export market for South Africa and is responsible for up to 46% of fresh fruit exports annually (Fruit South Africa, *2020 Key fruit statistics*). This market has zero-tolerance enforcement for the presence of non-EU Tephritidae, which includes all fruit fly pests in South Africa, except *C. capitata*, which is an established pest in the EU [14].

Therefore, it is necessary to accurately and reliably identify these five fruit fly species through surveillance practices prior to export. The primary goal of fruit fly management is to produce commercial fruit that are free of fruit flies. To facilitate this process, fruit fly management practices are applied before harvest, including pest monitoring, orchard sanitation, and the application of control products. A number of measures are applied after harvest, such as sorting, inspection, and, where necessary disinfestation treatments. Fruit fly surveillance programmes are also in place to detect the presence of exotic species such as *B. dorsalis* in pest-free areas in South Africa [15]. The success of these management practices is evaluated through routine monitoring and inspection programmes that provide estimates of population size and are useful in declaring pest-free zones. Surveillance programmes should accurately identify all fruit flies to species level [16]. Morphological keys for identifying adult fruit flies and third-instar larval specimens have been well developed [17, 18]. However, morphological identification becomes problematic when specimens are damaged, cryptic species are found, or early life stages are intercepted [3, 18–20]. When cases arise where species cannot be reliably distinguished through morphological methods, the use of molecular diagnostics would be more efficient.

DNA barcoding using cytochrome oxidase I (COI) has been used as a standard DNA marker for species identification. This molecular marker is relatively conserved within the same species, with variation present between different species allowing for identification [21]. This technique has been demonstrated to resolve most species; however, DNA barcoding becomes problematic when closely related and cryptic species are present where interspecific variation is reduced. COI of the fruit flies in this study have previously been investigated for species identification. These results were unable to resolve species complexes such as the *Ceratitis* FARQ complex (*Ceratitis fasciventris* (Bezzi), *Ceratitis anonae* Graham, *Ceratitis rosa*, and *Ceratitis quilicii*) and the *Bactrocera dorsalis* complex [3, 22–26]. A molecular assay that can differentiate between all five species simultaneously would be valuable for routine monitoring and pest surveillance. A multiplex assay has recently been developed for use at PoE in cases of larval interception [27]. While this assay is useful for time-sensitive identification matters, routine pest monitoring and surveillance may benefit from a sequencing-based assay as the resources are readily available at these facilities, and diversity seen within or between species can be studied further.

Non-coding and intergenic regions typically evolve at a faster rate than protein-coding genes. Thus, it is expected to see greater variation between species in these non-coding regions. Mitochondrial intergenic spacers have been targeted for use as species-specific markers in other organisms, including COI-COII intergenic region [28, 29], tRNA^leu^ – COII [30], tRNA^cys^ - tRNA^asn^ [31], atp6 – COX3 [32], tRNA^ile^ – tRNA^gln^ and, tRNA^gln^ – tRNA^met^ [33]. In this study, the mitochondrial intergenic region tRNA^ile^ – tRNA^gln^ (denoted as intergenic region I) and tRNA^gln^ – tRNA^met^ (denoted as intergenic region II) were amplified using a single primer pair and investigated for use as species-specific markers for the accurate identification of five tephritid fruit flies in South Africa.

## Results

### Species identity confirmation

Colony adult males and wild trap-collected specimens underwent morphological identification and identity confirmation using BLASTn analysis querying the COI gene, which was amplified using the primer pair CI-J2183 and TL2-N3014 [34]. All adult specimens used in this study were identified to species level through morphological identification using published keys [17], as well as molecular identification using a multiplex PCR previously developed for use at Ports of Entry [27]. COI could differentiate *C. capitata*, *C. cosyra* and *B. dorsalis* to species level. However, the high sequence similarity between *C. rosa*, *C. quilicii*, and the FARQ complex prevented the differentiation of these species. Although *C. capitata* and *Ceratitis caetrata* Munro share high sequence similarity in COI, the latter fly is not present in South Africa [35].

### Complete mitochondrial genome assembly and annotation

**Fig. 1:**
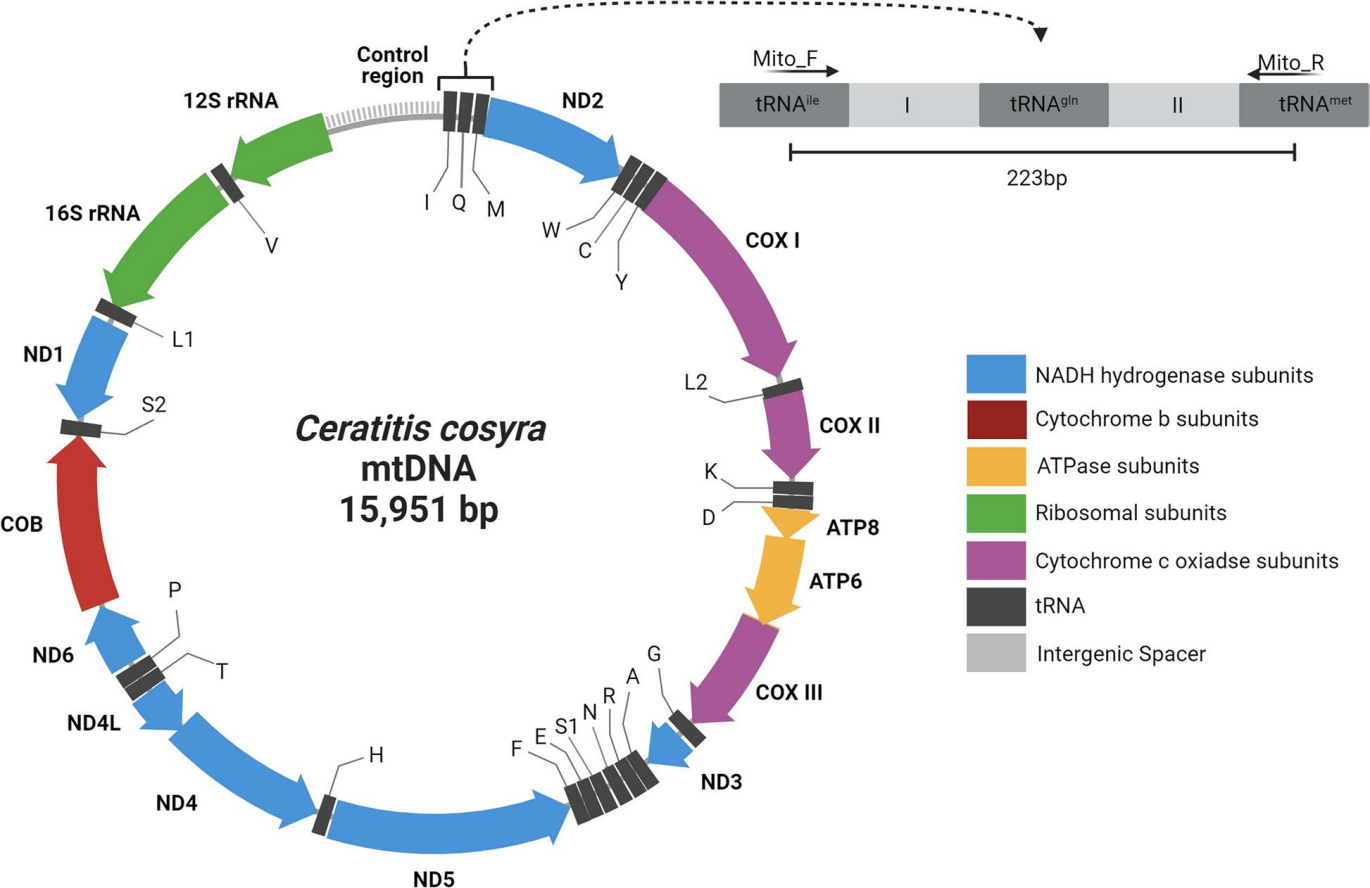
Schematic representation of the complete mitochondrial genome of *Ceratitis cosyra*. The top right corner is an enlarged schematic organisation of the amplicon generated by the primer pair Mito_F/R

An average of 165 932 970 reads per sample (STD = 4 195 444) were generated with high throughput sequencing (HTS). Mitogenomes assembled with CLC genomics workbench 11.0.1 (Qiagen) had an average read coverage of 7 740 (STD = 2 980.67). Mitogenomes assembled with MITObim [36] had an average coverage of 7 196 (STD = 2 714.31). The final accessions were generated with consensus sequences from an alignment of the two assembly methods. Variation between the two methods was resolved with Sanger sequencing. The complete mitochondrial genomes were high in similarity to existing references (Additional file 1). In this study, we assembled 10 complete mitochondrial genomes belonging to five different species in the genera *Ceratitis* and *Bactrocera* (Table 1). In total, 37 genes were annotated, including 13 protein-coding genes (PGCs), 22 tRNAs and two rRNAs (Fig. 1). All 10 mitochondrial genomes were highly similar in structural organisation, as previously described [22, 37–39].

**Table 1:** Mitochondrial genome assembly statistics for 10 fruit flies of the genera *Ceratitis* and *Bactrocera*, including NCBI GenBank database accessions

Specimen	Genome length (bp)	AT%	GC%	Accession
*Ceratitis capitata* 1	15,980	77.409	22.584	ON861815
*Ceratitis capitata* 2	15,981	77.417	22.583	ON861816
*Ceratitis cosyra* 1	15,954	76.194	23.806	ON861817
*Ceratitis cosyra* 2	15,951	76.158	23.842	ON861818
*Ceratitis quilicii* 1	16,020	77.197	22.803	ON861819
*Ceratitis quilicii* 2	16,028	77.396	22.604	ON861820
*Ceratitis rosa* 1	15,998	77.322	22.678	ON861821
*Ceratitis rosa* 2	15,998	77.316	22.684	ON861822
*Bactrocera dorsalis* 1	15,916	73.624	26.376	ON861823
*Bactrocera dorsalis* 2	15,915	73.616	26.384	ON861824

### Primer design

A single primer set, Mito_F/R, was designed to amplify a DNA fragment in all five fruit fly species under investigation (Table 2). Each PCR amplicon contained a partial sequence of tRNA^ile^ (56 bp), the complete sequence of tRNA^gln^ (69 bp), a partial sequence of tRNA^met^ (29 bp), and two intergenic spacers (Fig. 1). The sense primer is located between 12 and 36 bp (tRNA^ile^), and the antisense primer is located between 203 and 227 bp (tRNA^met^). Location refers to the *Bactrocera dorsalis* accession ON861824.

**Table 2:** Nucleotide sequence, location, and parameters of the primers designed in this study. ON861824 refers to the complete mitochondrial genome of *Bactrocera dorsalis 2*

Primer	Sequence 5’-3’	Location in ON861824	Tm (°C)	GC%
trnI_trnQ_F	TGAATTGCCTGACAAAAGGG	3–22	53.5	45.0
trnI_trnQ_R	GGTATGAACCCAGTAGCTTA	215–234	51.1	45.0
rrnS_trnI_F	GCTGGCACAAATTTAACCAA	14,787–14,806	52.0	40.0
rrnS_trnI_R	CCCTTTTGTCAGGCAATTCA	3–22	53.5	45.0
Mito_F	TGACAAAAGGGTTACCTTGATAGGG	12–36	56.6	44.0
Mito_R	ACCCAGTAGCTTAATTAGCTTATCT	203–227	53.4	36.0

### High-resolution melt analysis

The primer set Mito_F/R used in this study was initially designed for use in a high-resolution melt (HRM) for species differentiation based on melting point analysis of the resulting amplicon. The average melt temperature (Tm) and standard deviation of each species-specific amplicon are presented in Table 3. However, this approach was unsuccessful due to the low GC content of the selected region in the mitochondrial genome affecting the consistency of dye intercalation. Hence, melt-point intervals were inconsistent within species, and confidence intervals could not be accurately determined.

**Table 3:** Average melt temperature (Tm) and standard deviation of each fruit fly species-specific amplicon based on high-resolution melt analysis of amplicon Mito_F/R

Species	Average Tm (°C)	Standard deviation
*C. capitata*	72.46	0.63
* C. cosyra*	72.77	0.18
* C. quilicii*	71.99	0.45
* C. rosa*	72.06	0.31
*B. dorsalis*	73.98	0.39

### Sequence analysis

**Fig. 2:**
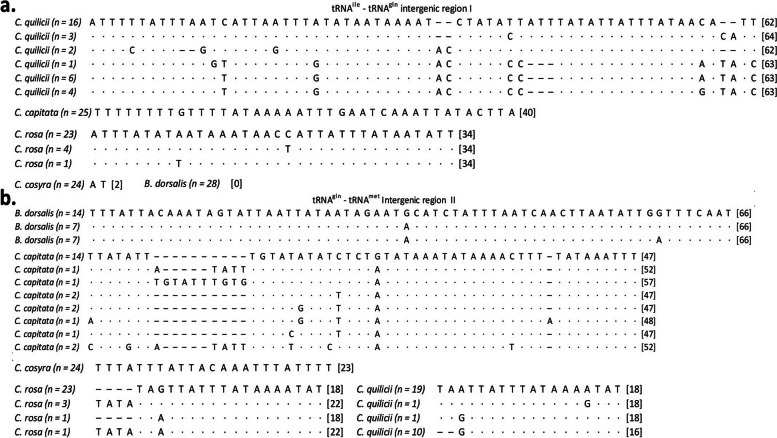
Nucleotide sequence comparison of intergenic regions I (**a**) and II (**b**) showing the variation present within each species and the difference in the size of intergenic regions between each species. Dashes represent a gap or missing nucleotide, and dots represent a matching nucleotide. The number of specimens with a particular intergenic sequence is indicated in round brackets next to the species name, and the size of the intergenic region is indicated in square brackets at the end of each nucleotide sequence

A total of 20 specimens per species underwent PCR and amplicon sequencing. Multiple sequence alignments of the amplicons generated by primer set Mito_F/R and available reference sequences demonstrate the ability to differentiate between the five fruit fly species based on intergenic regions (Fig. 2). BLASTn analysis of the whole Mito_F/R amplicon against the NCBI GenBank database highlighted high sequence similarity between *C. quilicii*, *C. fasciventris* (100%), and *C. anonae* (97.78%), between *C. cosyra*, *Ceratitis pallidula* De Meyer, Mwatawala & Virgilio (97.04%), and *Ceratitis quinaria* (Bezzi) (96.45%), between *B. dorsalis, Bactrocera invadens* Drew, Tsuruta & White (99.51%), *Bactrocera carambolae* Drew & Hancock (99.51%), *Bactrocera philippinensis* Drew & Hancock (99.51%), *Bactrocera papayae* Drew & Hancock (99.51%), *Bactrocera ruiliensis* Wang, Long & Zhang (97.57%), and *Bactrocera thailandica* Drew and Romig (97.57%). Local BLASTn analyses which queried the amplicons generated by the primer set Mito_F/R against a dataset containing only mitochondrial genomes of *C. capitata*, *C. cosyra*, *C. quilicii*, *C. rosa*, and *B. dorsalis*, identified each amplicon to the relevant species accurately. More specifically each of the five fruit fly species investigated in this study can be differentiated by the size of intergenic region I (tRNA^ile^ – tRNA^gln^). Intergenic region II (tRNA^gln^ - tRNA^met^) does not allow for unambiguous differentiation of the five fruit fly species on either size differences or multiple sequence alignment.

## Discussion

The generation of complete mitochondrial genome sequences adds value to publicly available online databases by providing increasingly extensive resources. Complete mitochondrial genomes provide useful molecular markers for both taxonomic and molecular studies. These markers have been widely used in the study of insects and Tephritidae in particular [40–43]. The availability and abundance of complete mitochondrial genomes are essential for studying closely related and cryptic species, specifically when existing species identification tools are limited in efficacy.

While the HRM analysis was unable to consistently differentiate between species, the nucleotide sequences generated from these amplicons were applicable in the species identification of the five fruit flies. The variation in size of the complete mitochondrial genome between members of the family Tephritidae is mainly due to variation in non-coding regions and intergenic spacers [44]. Mitochondrial intergenic regions, including intergenic region I between tRNA^ile^ and tRNA^gln^, have previously been utilised in analyses of phylogeny and genetic distance [33]. The length of intergenic region I is a potential tool for differentiation of these five species, *C. capitata* (40 bp), *C. cosyra* (2 bp), *C. quilicii* (62–64 bp), *C. rosa* (34 bp), and B. dorsalis (0 bp) (Fig. 2). The absence of intergenic region I in *B. dorsalis* has previously been described and is common among members of the genus *Bactrocera* [39, 44, 45]. Due to high similarity, intergenic region II could not differentiate between *C. rosa* and *C. quilicii* (Fig. 2). This high similarity is in both sequence identity and the size of intergenic region II; *C. quilicii* (16–18 bp), *C. rosa* (18–22 bp). Furthermore, this region showed greater variation between individuals of the same species than intergenic region I, specifically in the case of *C. capitata*.

BLASTn analysis of amplicons generated with Mito_F/R against a local database consisting of the mitochondrial genomes of only the five fruit fly species investigated in this study accurately differentiates and identifies these flies to species level. In comparison, BLASTn analysis of the same amplicons against the NCBI database revealed several potential confounding species. None of the species identified are currently present or have ever been reported in South Africa [2, 46, 47], with the exception being *C. quinaria*. However, this fly is not regarded as a pest of economic importance in commercial fruit in South Africa. Three fruit fly species confounding the BLAST analysis of the *B. dorsalis* Mito_F/R amplicon have been synonymised as *B. dorsalis*, namely, *B. invadens*, *B. philippinensis*, and *B. papayae* [48]. The *C. quilicii* Mito_F/R amplicon is highly similar to *C. fasciventris* (96.90–100%) and *C. anonae* (93.81–97.87%). However, the amplicon does not share a high similarity with that of *C. rosa* (84.44%). This allows for unambiguous differentiation of the cryptic species *C. quilicii* and *C. rosa*, which cannot be differentiated based on COI due to high sequence similarity within the *Ceratitis* FARQ complex [3, 22, 23].

Interestingly, *C. rosa* did not share a high similarity with the other members of the FARQ complex within the Mito_F/R amplicon region. High similarity of the amplicon between *C. quilicii*, *C. fasciventris*, and *C. anonae* corresponds with previous studies where evidence of gene flow between *C. quilicii* and *C. fasciventris* suggests an admixture event between these two species [22, 49]. In addition, it has been reported that *C. fasciventris* and *C. quilicii* or *C. rosa*, which were previously recognised as a single species [3], can reproduce under laboratory conditions [50].

Variation in noncoding regions is not uncommon as intergenic spacers can differ considerably, even in the case of closely related species. Notably in this study, the size of the intergenic region I is relatively consistent within species and significantly different between species to allow for unambiguous differentiation of these five fruit fly species. Species identification based on the size of mitochondrial intergenic regions has shown to be successful in a wide range of bacteria and is emerging in insect phylogenetics [33, 51–54]. It is important to frame this work in a greater context of mitochondrial datasets being at the forefront of the exploration of evolutionary relationships. An advantage of the identification process described is that the primer set used is universal as the target regions are located within conserved tRNA’s. In principle, this tool has the potential to study multiple genera within the family Tephritidae.

This study reports 10 complete mitochondrial genomes for the fruit flies *C. capitata*, *C. cosyra*, *C. rosa*, *C. quilicii* and *B. dorsalis*. The availability of these mitogenomes will aid future studies regarding tephritid fruit flies. We propose that the length of intergenic region I between tRNA^ile^ and tRNA^gln^, and multiple sequence alignment of the amplicon Mito_F/R against reference sequences can be used as informative species-specific markers for differentiation of these five tephritid flies present in South Africa. The identification tool described in this study can be used as an alternative to traditional DNA barcoding for accurate species identification of these flies for routine pest monitoring practices in South Africa. Furthermore, the inherent impact of studying intergenic spacers such as those described in this study offers advancement and utility in global fruit fly research and diversity studies, and can potentially be expanded for phylogenetic and taxonomic evaluation.

## Methods and materials

### Sample collection

Colony insects and larvae were provided by Citrus Research International (CRI) in Mbombela, Mpumalanga, South Africa, from established colonies (Additional file 2). Confirmation of the identities of fruit fly species in the colonies (adult specimens from colonies refreshed in the period 2020–2021) was performed by Marc De Meyer, Royal Museum for Central Africa, on 21 February 2022. Wild, male, fruit fly specimens used in this study were collected from traps (Additional file 3). *Ceratitis* flies were trapped with McPhail type bucket traps baited with enriched ginger root oil (EGO lure) (Insect Science, Tzaneen, South Africa), and *B. dorsalis* flies were trapped with Chempac bucket traps baited with methyl eugenol (ME) (Invader lure, River Bioscience, Gqeberha, South Africa). Fruit fly specimens were maintained in 100% ethanol at 4 °C until processed.

### DNA extraction and species identification

DNA extracts used for high throughput sequencing were obtained from single, adult-male colony insects following an adapted protocol by Sunnucks and Hales [55], with TNES buffer (50 mM Tris, pH 7.5, 400 mM NaCl, 20 mM EDTA, 0.5% SDS) substituted for 180 µl ATL buffer (Qiagen). Incubation time was lengthened overnight at 56 °C. RNase A was added to the supernatant after NaCl precipitation, and the second precipitation was performed with ice-cold 100% isopropanol overnight at -20 °C. DNA concentration and quality were quantified using a NanoDrop 2000 spectrophotometer and a Qubit dsDNA BR assay kit (Invitrogen). Total DNA was also extracted for PCR from colony adults, colony larvae, and wild, trap-collected insects following the destructive protocol of the DNeasy Blood and Tissue Kit (Qiagen), where the whole body of the fruit fly was used.

Each adult colony male specimen in this study underwent molecular identification using the universal primer set CI-J2183 and TL2-N301434 for amplification and Sanger sequencing of the COI gene [34]. The PCR was performed in a total volume of 25 µl containing 1x Kapa Taq buffer A (KAPA Biosystems), 0.2 mM dNTP mix (Thermo Scientific), 0.4 µM of each primer (CI-J2183 and TL2-N3014), and 0.05 U KAPA Taq DNA Polymerase (KAPA Biosystems). Cycling conditions included an initial denaturation step at 94 °C for 5 min, followed by 35 cycles of 94 °C for 30 s, annealing at 50 °C for 30 s and extension at 72 °C for 45 s. The final extension was performed at 72 °C for 7 min.

All specimens in this study, colony larvae, colony adults, and wild adults underwent identification using a multiplex PCR assay developed for the identification of these five fruit fly species of economic importance to South Africa following the protocol outlined in the study [27].

### High throughput sequencing

Two DNA extracts per species were sent for high throughput sequencing at Macrogen (South Korea). Library construction and high throughput sequencing of the colony insects were performed by Macrogen on the Illumina NovaSeq 6000 platform (2 × 150 bp paired-end reads). The TruSeq DNA PCR-Free Kit was used for library preparation of the samples *C. rosa* 2, *C. quilicii* 1 & 2 and *C. cosyra* 2; and the TruSeq Nano DNA Kit was used for library preparation of samples *C. capitata* 1 & 2, *C. rosa* 1, *C. cosyra* 1, and *B. dorsalis* 1 & 2.

### Mitogenome assembly and annotation

Sequencing reads were quality checked using FastQC, all reads and bases were of good quality and further quality checking or trimming was not required. Reference-based assembly was performed with MITObim [36] using *Ceratitis fasciventris* (GenBank accession NC_035497.1) [56] as a reference template. Assembly was implemented with the following parameters; job = genome, mapping accurate, technology = solexa, parameters=-NW:cmrnl = war, start < 1>, end < 30>. De novo assembly was performed in CLC genomics workbench version 11.0.1 (Qiagen) using the parameters; automatic bubble size, automatic word size, map reads back to contigs (slow), minimum contig length = 200, mismatch cost = 2, insertion cost = 3, deletion cost = 3, Length fraction = 0.5, and similarity fraction = 0.8. The CLC de novo assembled mitogenome and reference-based assembly for each specimen were aligned, and regions with discrepancies between the two methods were validated with Sanger sequencing. The consensus sequence taken from the alignment of the two assembled mitogenomes and validated variable regions for each specimen was used for manual curation. Manual curation was performed by aligning consensus sequences to relevant reference sequences, *C. capitata* (NC_000857.1) [37], *C. cosyra* (MT036783.1), *C. quilicii*, (MT998948.1), *C. rosa* (MT997010.1) [22] and *B. dorsalis* (KT343905.1), to confirm the starting and ending point of each mitogenome. Mitochondrial genome annotations were performed using the MITOs web server with the parameter “genetic code: 05 – invertebrate” [57] and checked by manually translating the coding domains.

### Validation of variable regions

The mitochondrial intergenic region between tRNA^ile^ and tRNA^gln^ was validated using the primer pair trnI_trnQ_F/R. The non-coding region at the 3’end of the genome, known as the control region (CR) and origin of replication (Fig. 1), was validated using the primer pair rrnS_trnI_F/R (Table 2). Both primer sets were designed with Oligo Explorer 1.1.2 (Gene Link) and synthesised by IDT. PCR reactions were performed in a total volume of 25 µl, with 1x Kapa Taq buffer A (Kapa Biosystems), 0.2 mM dNTP mix (Thermo Scientific), 0.4 µM of each primer (trnI-trnQ _F/R or rrnS_trnI_F/R), 0.05 U KAPA Taq DNA Polymerase (Kapa Biosystems). Cycling consisted of initial denaturation at 94 °C for 5 min, with 30 cycles of 94 °C for 30 s, annealing at 50 °C for 30 s, extension at 72 °C for 30 s (primer set trnI_trnQ_F/R), or 72˚C for 1 min and 20 s (primer set rrnS_trnI_F/R). The final extension for both primer sets was performed at 72 °C for 7 min.

PCR products were visualised on 2% agarose-TAE gels, purified using the Zymoclean Gel DNA Recovery Kit (Zymo Research), and sent for bidirectional Sanger sequencing at the Central Analytical Facilities at Stellenbosch University.

### Primer design and species differentiation

The complete mitochondrial genomes of 30 specimens (Additional file 4) belonging to the five species of interest were downloaded from the publicly available NCBI GenBank database and aligned with the 10 complete mitochondrial genomes assembled in this study using CLC Genomics workbench 11.0.1 (Qiagen). Sites with variability between species were visually identified. One primer set (IDT), Mito_F/Mito_R, was designed to amplify mitochondrial DNA between tRNA^ile^ and tRNA^met^ (Fig. 1) using Oligo Explorer 1.1.2 (GeneLink) (Table 2). Five adult colony insects, five colony larvae, and 10 wild, trap-collected insects per species were subjected to PCR. PCRs were performed on a Qiagen Rotor-Gene Q thermal cycler. Each reaction contained 1X Kapa Taq Buffer A (Kapa Biosystems), 0.4 µM forward primer (IDT), 0.4 µM reverse primer (IDT), 0.2 mM dNTP mix (Thermo Scientific), 1.5 µM SYTO-9 (Invitrogen), 0.05 U KAPA Taq (Kapa Biosystems) and 100 ng DNA. Cycling was conducted on a 36-well carousel with auto gain optimisation performed before the first acquisition initial hold at 94 °C for 5 min, followed by 45 cycles of 94 °C for 30 s, annealing at 55˚C for 30 s, and extension at 72 °C for 30 s. High-resolution melting curves of the PCR amplicons were obtained with temperatures ranging from 70 to 90 °C, with a 0.1 °C increase in temperature every two seconds. HRM curve analysis was performed with Rotor-Gene Q software version 2.3.5 (Qiagen). Amplicons were visualised on a 2% agarose gel to assess specificity (Additional file 5), and bi-directionally Sanger sequenced at the Central Analytical Facilities at Stellenbosch University for downstream sequence analysis.

### Sequence analysis

Multiple sequence alignments of the Mito_F/R amplicon were conducted in CLC genomics workbench version 11.0.1 (Qiagen). The alignment consisted of the 40 complete mitochondrial genomes utilised for primer design and 20 amplicon sequences per species (Fig. 2). Amplicons were queried against the NCBI BLASTn database to identify any confounding species with a high sequence similarity to the queried specimens. The amplicons were also queried against a local BLASTn database created on CLC genomics workbench version 11.0.1 (Qiagen), consisting of only the mitochondrial genomes of the five fruit fly species of concern in this study.

## Supplementary Information

**Additional file 1.** BLASTn results ascertained from querying the ten mitogenomes generated in this study against the publicly available GenBank online database. Table demonstrates high similarity between the query genomes generated in this study and the closest hit to members of the same species available in the GenBank database.

**Additional file 2.**  Collection information of colony flies and respective larvae reared at CRI (Mbombela, Mpumalanga, South Africa). Initial collection sites of the established colonies are provided as coordinates. Adult colony insects were collected in February 2021; these colonies were refreshed between January 2019 and January 2020. Larval specimens were collected in August 2021; these colonies were refreshed between November 2020 and May 2021. 

**Additional file 3.** Sample collection data for the wild, trap-collected male specimens used for assay validation in this study. The collection site is provided as the province and coordinates.

**Additional file 4.** List of complete mitochondrial genomes available in the NCBI GenBank database used for primer design and multiple sequence comparison.

**Additional file 5.** 2% agarose TAE gel visualised with ethidium bromide displaying specificity of the primer pair Mito_F/R. Lane 1: C. capitata, Lane 2: C. cosyra, Lane 3: C. quilicii, Lane 4: C. rosa, Lane 5: B. dorsalis, Lane 6: no template control, Lane L: 100 bp DNA ladder (Thermo Scientific). Amplicon sizes are indicated on the gel. 

## Data Availability

The datasets used and/or analysed during the current study are available on the NCBI GenBank database under the accessions ON861815 - ON861824.
