# Supplementary material for: Utilisation of a mitochondrial intergenic region for species differentiation of fruit flies (Diptera: Tephritidae) in South Africa
Source: BMC Genomics. 2022 Dec 1;23:793. doi: 10.1186/s12864-022-09038-x (PMC9716763; doi:10.1186/s12864-022-09038-x)
Supplement: Supplementary file 1 — Additional file 1. BLASTn results ascertained from querying the ten mitogenomes generated in this study against the publicly available GenBank online database. Table demonstrates high similarity between the query genomes generated in this study and the closest hit to members of the same species available in the GenBank database. [file 12864_2022_9038_MOESM1_ESM.docx]

**Utilisation of a mitochondrial intergenic region for species differentiation of fruit flies (Diptera: Tephritidae) in South Africa**

**Kelsey J Andrews^1^, Rachelle Bester^1,2^, Aruna Manrakhan^3,4^, and Hans J Maree^1,2,*^**

^1^Department of Genetics, Stellenbosch University, Private Bag X1, Matieland, 7602, South Africa

^2^Citrus Research International, PO Box 2201, Matieland, 7602, South Africa

^3^Citrus Research International, PO Box 28, Mbombela, 1200, South Africa

^4^Department of Conservation Ecology and Entomology, Stellenbosch University, Private Bag X1, Matieland 7602, South Africa

[*hjmaree@sun.ac.za](mailto:*hjmaree@sun.ac.za)

**Additional file 1:** BLASTn results ascertained from querying the ten mitogenomes generated in this study against the publicly available GenBank online database. Table demonstrates the high similarity between the query genomes generated in this study and the closest hit to members of the same species available in the GenBank database.

| Specimen | Hit | Accession | E-value | % Identity |
| --- | --- | --- | --- | --- |
| *C. capitata* (1) | *Ceratitis capitata* | NC_000857.1 | 0.0 | 99.74 |
| *C. capitata* (2) | *Ceratitis capitata* | NC_000857.1 | 0.0 | 99.74 |
| *C. cosyra* (1) | *Ceratitis cosyra* | MT036784.1 | 0.0 | 99.50 |
| *C. cosyra* (2) | *Ceratitis cosyra* | MT036784.1 | 0.0 | 99.20 |
| *C. rosa* (1) | *Ceratitis rosa* | MT036799.1 | 0.0 | 99.87 |
| *C. rosa* (2) | *Ceratitis rosa* | MT036799.1 | 0.0 | 99.87 |
| *C. quilicii* (1) | *Ceratitis quilicii* | MT036776.1 | 0.0 | 99.91 |
| *C. quilicii* (2) | *Ceratitis quilicii* | MT036778.1 | 0.0 | 99.67 |
| *B. dorsalis* (1) | *Bactrocera dorsalis* | MN104220.1 | 0.0 | 99.86 |
| *B. dorsalis* (2) | *Bactrocera dorsalis* | MN104220.1 | 0.0 | 99.87 |
