# Supplementary material for: Utilisation of a mitochondrial intergenic region for species differentiation of fruit flies (Diptera: Tephritidae) in South Africa
Source: BMC Genomics. 2022 Dec 1;23:793. doi: 10.1186/s12864-022-09038-x (PMC9716763; doi:10.1186/s12864-022-09038-x)
Supplement: Supplementary file 2 — Additional file 2. Collection information of colony flies and respective larvae reared at CRI (Mbombela, Mpumalanga, South Africa). Initial collection sites of the established colonies are provided as coordinates. Adult colony insects were collected in February 2021; these colonies were refreshed between January 2019 and January 2020. Larval specimens were collected in August 2021; these colonies were refreshed between November 2020 and May 2021. [file 12864_2022_9038_MOESM2_ESM.docx]

**Utilisation of a mitochondrial intergenic region for species differentiation of fruit flies (Diptera: Tephritidae) in South Africa**

**Kelsey J Andrews^1^, Rachelle Bester^1,2^, Aruna Manrakhan^3,4^, and Hans J Maree^1,2,*^**

^1^Department of Genetics, Stellenbosch University, Private Bag X1, Matieland, 7602, South Africa

^2^Citrus Research International, PO Box 2201, Matieland, 7602, South Africa

^3^Citrus Research International, PO Box 28, Mbombela, 1200, South Africa

^4^Department of Conservation Ecology and Entomology, Stellenbosch University, Private Bag X1, Matieland 7602, South Africa

[*hjmaree@sun.ac.za](mailto:*hjmaree@sun.ac.za)

**Additional file 2:** Collection information of colony flies and respective larvae reared at CRI (Mbombela, Mpumalanga, South Africa). Initial collection sites of the established colonies are provided as coordinates. Adult colony insects were collected in February 2021; these colonies were refreshed between January 2019 and January 2020. Larval specimens were collected in August 2021; these colonies were refreshed between November 2020 and May 2021.

| Life stage | Species | Rearing fruit | | Collection Date | Latitude | Longitude |
| --- | --- | --- | --- | --- | --- | --- |
| Adult male | *C. capitata* | Coffee | (*Coffea canephora Pierre ex. Froehner)* | 10/03/2019 | 31°5’15.63” E | 25°6’43.50” S |
|  | *C. cosyra* | Marula | (*Sclerocarya birrea* (A. Rich.) Hochst.) | 1/29/2019 | 31°2’35.70” E | 25°28’4.03” S |
|  | *C. quilicii* | Peach | (*Prunus persica* L. Batsch) | 1/21/2020 | 30°23’34.73” E | 24°59’47.17” S |
|  | *C. rosa* | Strawberry Guava | (*Psidium cattleyanum* Sabine) | 11/13/2019 | 30°58’10.99’’ E | 25°27’08.54’’ S |
|  | *B. dorsalis* | Mango | (*Mangifera indica* L.) | 1/29/2019 | 30°58’10.99’’ E | 25°27’08.54’’ S |
| Larvae | *C. capitata* | Coffee | (*Coffea canephora Pierre ex.* Froehner) | 3/10/2021 | 31°5’15.63” E | 25°6’43.50” S |
|  | *C. cosyra* | Pepper-bark tree | (*Warburgia salutaris* (Bertol.f.) Chiov.) | 11/30/2020 | 30°58’6.10” E | 25°26’37.92” S |
|  | *C. quilicii* | Pineapple guava | (*Feijoa sellowiana* (O.Berg) O.Berg) | 05/2021 | 29°59’ 11.56” E | 26°30’51.31” S |
|  | *C. rosa* | Jambos | (*Syzygium jambos* L. Alston) | 11/27/2020 | 30°58’10.99’’ E | 25°27’08.54’’ S |
|  | *B. dorsalis* | Mango | (*Mangifera indica* L.) | 2/12/2021 | 30°57’ 15.84” E | 25°32’58.06” S |
