# Supplementary material for: Utilisation of a mitochondrial intergenic region for species differentiation of fruit flies (Diptera: Tephritidae) in South Africa
Source: BMC Genomics. 2022 Dec 1;23:793. doi: 10.1186/s12864-022-09038-x (PMC9716763; doi:10.1186/s12864-022-09038-x)
Supplement: Supplementary file 3 — Additional file 3. Sample collection data for the wild, trap-collected male specimens used for assay validation in this study. The collection site is provided as the province and coordinates. [file 12864_2022_9038_MOESM3_ESM.docx]

**Utilisation of a mitochondrial intergenic region for species differentiation of fruit flies (Diptera: Tephritidae) in South Africa**

**Kelsey J Andrews^1^, Rachelle Bester^1,2^, Aruna Manrakhan^3,4^, and Hans J Maree^1,2,*^**

^1^Department of Genetics, Stellenbosch University, Private Bag X1, Matieland, 7602, South Africa

^2^Citrus Research International, PO Box 2201, Matieland, 7602, South Africa

^3^Citrus Research International, PO Box 28, Mbombela, 1200, South Africa

^4^Department of Conservation Ecology and Entomology, Stellenbosch University, Private Bag X1, Matieland 7602, South Africa

[*hjmaree@sun.ac.za](mailto:*hjmaree@sun.ac.za)

**Additional file 3:** Sample collection data for the wild, trap-collected male specimens used for assay validation in this study. The collection site is provided as the province and coordinates.

| Species | Collection date | Province | Coordinates |
| --- | --- | --- | --- |
| *B. dorsalis* | 08/2021 | Mpumalanga | 31°04’17.41” E 25°26’38.27” S |
|  | 07/2021 | Mpumalanga | 30°34’31.48” E 25°23’52.07” S |
|  | 06/2021 | Limpopo | 30°50’51.95” E 24°24’26.61” S |
|  | 09/2021 | Limpopo | 30°32’36.02” E 23°45’18.95” S |
|  | 06/2021 | Limpopo | 30°22’54.60” E 23°52’21.32” S |
| *C. quilicii* | 06/2021 | Northwest | 25°46’12.9” S  27°36’51.7” E |
|  | 06/2021 | Mpumalanga | 25°22’50.7” S  30°32’02.2” E |
|  | 06/2021 | KwaZulu Natal | 27°21’44.7” S  31°47’14.4” E |
|  | 03/2021 | Free State | 28°15’09.1”S  28°19’02”E |
|  | 06/2021 | Eastern Cape | 33°36’43.4”S  25°39’39.1”E |
| *C. capitata* | 06/2021 | Northwest | 25°46’12.9”S  27°36’51.7”E |
|  | 06/2021 | Limpopo | 23°51’47.7”S  30°23’08.4”E |
|  | 06/2021 | Mpumalanga | 25°26’39.3”S  31°33’15.4”E |
|  | 06/2021 | KwaZulu Natal | 27°21’44.7”S  31°47’ 14.4”E |
|  | 05/2021 | Northern Cape | 28°48’8.83”S  20°39’56.2”E |
| *C. cosyra* | 06/2021 | Northwest | 25°46’12.9”S  27°36’51.7”E |
|  | 06/2021 | Limpopo | 23°51’47.7”S  30°23’08.4”E |
|  | 06/2021 | Mpumalanga | 25°24’34.3”S  30°55’46.6”E |
|  | 08/2021 | KwaZulu Natal | 27°21’44.7”S  31°47’14.4”E |
| *C. rosa* | 06/2021 | Mpumalanga | 25°26’44.9”S  30°58’05.1”E |
