# Supplementary material for: Utilisation of a mitochondrial intergenic region for species differentiation of fruit flies (Diptera: Tephritidae) in South Africa
Source: BMC Genomics. 2022 Dec 1;23:793. doi: 10.1186/s12864-022-09038-x (PMC9716763; doi:10.1186/s12864-022-09038-x)
Supplement: Supplementary file 4 — Additional file 4. List of complete mitochondrial genomes available in the NCBI GenBank database used for primer design and multiple sequence comparison. [file 12864_2022_9038_MOESM4_ESM.docx]

**Utilisation of a mitochondrial intergenic region for species differentiation of fruit flies (Diptera: Tephritidae) in South Africa**

**Kelsey J Andrews^1^, Rachelle Bester^1,2^, Aruna Manrakhan^3,4^, and Hans J Maree^1,2,*^**

^1^Department of Genetics, Stellenbosch University, Private Bag X1, Matieland, 7602, South Africa

^2^Citrus Research International, PO Box 2201, Matieland, 7602, South Africa

^3^Citrus Research International, PO Box 28, Mbombela, 1200, South Africa

^4^Department of Conservation Ecology and Entomology, Stellenbosch University, Private Bag X1, Matieland 7602, South Africa

[*hjmaree@sun.ac.za](mailto:*hjmaree@sun.ac.za)

**Additional file 4:** List of complete mitochondrial genomes available in the NCBI GenBank database used for primer design and multiple sequence comparison.

| Species | Accession number |
| --- | --- |
| *Ceratitis quilicii* | MT998948.1 |
|  | MT036776.1 |
|  | MT036777.1 |
|  | MT036778.1 |
|  | MT036779.1 |
|  | MT036780.1 |
|  | MT036791.1 |
|  | MT036792.1 |
|  | MT036793.1 |
|  | MT036794.1 |
|  | MT036795.1 |
|  | MT036790.1 |
|  | NC_053846.1 |
| *Ceratitis rosa* | MT036796.1 |
|  | MT036797.1 |
|  | MT036798.1 |
|  | MT036799.1 |
|  | MT036800.1 |
|  | MT997010.1 |
| *Ceratitis capitata* | AJ242872.1 |
|  | NC_000857.1 |
|  | MT036782.1 |
| *Ceratitis cosyra* | MT036783.1 |
|  | MT036784.1 |
| *Bactrocera dorsalis* | KT343905.1 |
|  | DQ917577.1 |
|  | DQ845759.1 |
|  | NC_008748.1 |
|  | MG916968.1 |
|  | MN104220.1 |
