# Supplementary material for: Utilisation of a mitochondrial intergenic region for species differentiation of fruit flies (Diptera: Tephritidae) in South Africa
Source: BMC Genomics. 2022 Dec 1;23:793. doi: 10.1186/s12864-022-09038-x (PMC9716763; doi:10.1186/s12864-022-09038-x)
Supplement: Supplementary file 5 — Additional file 5. 2% agarose TAE gel visualised with ethidium bromide displaying specificity of the primer pair Mito_F/R. Lane 1: C. capitata, Lane 2: C. cosyra, Lane 3: C. quilicii, Lane 4: C. rosa, Lane 5: B. dorsalis, Lane 6: no template control, Lane L: 100 bp DNA ladder (Thermo Scientific). Amplicon sizes are indicated on the gel. [file 12864_2022_9038_MOESM5_ESM.docx]

**Utilisation of a mitochondrial intergenic region for species differentiation of fruit flies (Diptera: Tephritidae) in South Africa**

**Kelsey J Andrews^1^, Rachelle Bester^1,2^, Aruna Manrakhan^3,4^, and Hans J Maree^1,2,*^**

^1^Department of Genetics, Stellenbosch University, Private Bag X1, Matieland, 7602, South Africa

^2^Citrus Research International, PO Box 2201, Matieland, 7602, South Africa

^3^Citrus Research International, PO Box 28, Mbombela, 1200, South Africa

^4^Department of Conservation Ecology and Entomology, Stellenbosch University, Private Bag X1, Matieland 7602, South Africa

[*hjmaree@sun.ac.za](mailto:*hjmaree@sun.ac.za)

**Additional file 5**


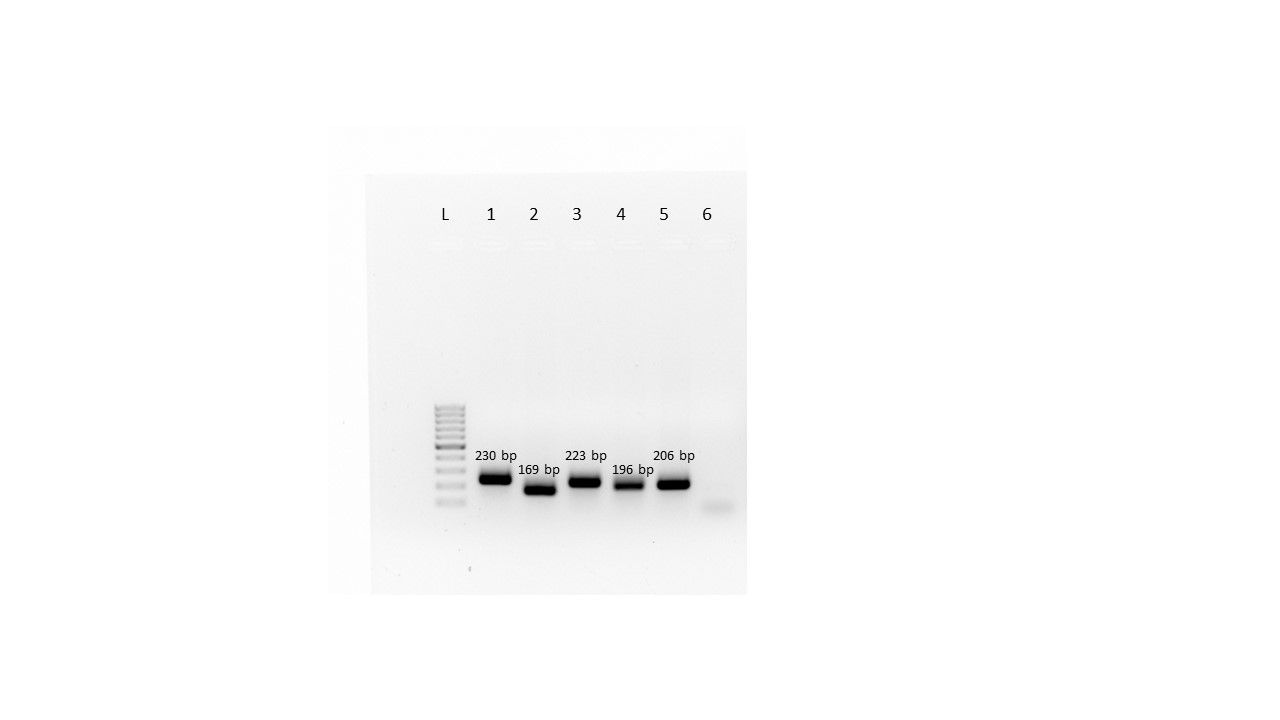
 **Additional file 5:** 2% agarose TAE gel visualised with ethidium bromide displaying specificity of the primer pair Mito_F/R. Lane 1: *C. capitata*, Lane 2: *C. cosyra*, Lane 3: C*. quilicii*, Lane 4: *C. rosa*, Lane 5: *B. dorsalis*, Lane 6: no template control, Lane L: 100 bp DNA ladder (Thermo Scientific). Amplicon sizes are indicated on the gel.
